# Supplementary material for: West Nile virus transmission potential in Portugal
Source: Commun Biol. 2022 Jan 10;5:6. doi: 10.1038/s42003-021-02969-3 (PMC8748923; doi:10.1038/s42003-021-02969-3)
Supplement: Supplementary file 3 — Supplementary Information [file 42003_2021_2969_MOESM3_ESM.pdf]

West Nile virus transmission potential in Portugal

Authors

José Lourenço [1,2*], Sílvia C Barros [3], Líbia Zé-Zé [4,2], Daniel SC Damineli [5], Marta Giovanetti [6,7], Hugo C Osório [4,8], Fátima Amaro [4,8], Ana M Henriques [3], Fernanda Ramos [3], Tiago Luís [3], Margarida D Duarte [3], Teresa Fagulha [3], Maria J Alves [4,8], Uri Obolski [9,10]

Affiliations

1 *Department of Zoology, University of Oxford, Oxford, United Kingdom*
2 *Biosystems and Integrative Sciences Institute, Edificio TecLabs, Campus da FCUL, Lisboa, Portugal*
3 *Instituto Nacional de Investigação Agrária e Veterinária, Virology Laboratory, Oeiras, Portugal*
4 *Centro de Estudos de Vectores e Doenças Infecciosas, Instituto Nacional de Saúde Doutor Ricardo Jorge, Marateca, Portugal*
5 *Department of Pediatrics, Faculdade de Medicina da Universidade de São Paulo, São Paulo, Brazil*
6 *Laboratório de Flavivírus, Instituto Oswaldo Cruz Fiocruz, Rio de Janeiro, Brazil*
7 *Laboratório de Genética Celular e Molecular, Universidade Federal de Minas Gerais, Minas Gerais, Brazil*
8 *Instituto de Saúde Ambiental, Faculdade de Medicina da Universidade de Lisboa, Lisboa, Portugal*
9 *School of Public Health, Faculty of Medicine, Tel Aviv University, Tel Aviv, Israel*
10 *Porter School of the Environment and Earth Sciences, Faculty of Exact Sciences, Tel Aviv University, Tel Aviv, Israel*

** Corresponding author(s): José Lourenço, jose.lourenco@zoo.ox.ac.uk*

Supplementary Figures 1-2 and Supplementary Tables 1-3.

Supplementary Figures

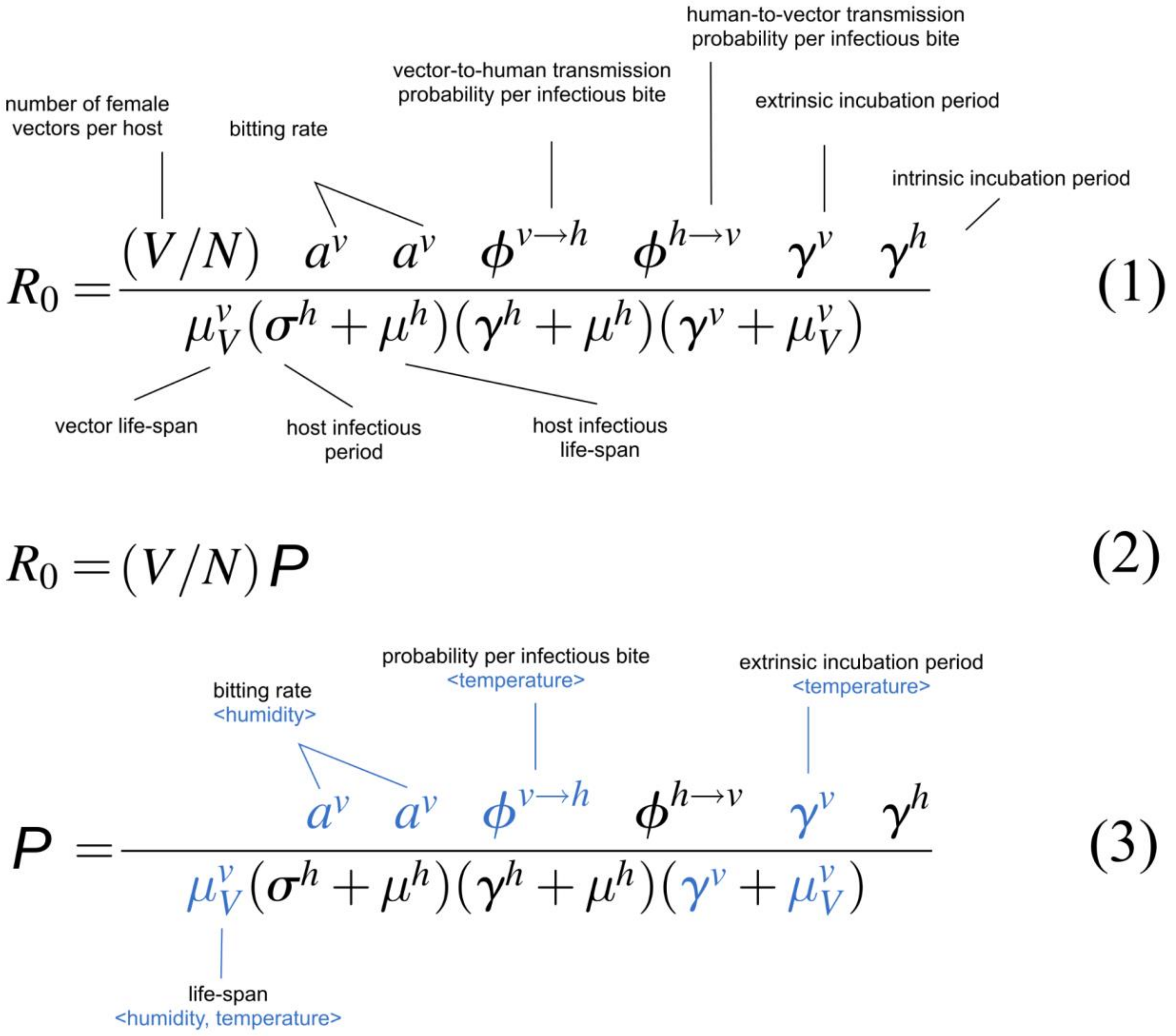

$$R_0 = \frac{(V/N)\ a^v\ a^v\ \phi^{v\to h}\ \phi^{h\to v}\ \gamma^v\ \gamma^h}{\mu_V^v(\sigma^h+\mu^h)(\gamma^h+\mu^h)(\gamma^v+\mu_V^v)} \quad (1)$$

$$R_0 = (V/N)\,P \quad (2)$$

$$P = \frac{a^v\ a^v\ \phi^{v\to h}\ \phi^{h\to v}\ \gamma^v\ \gamma^h}{\mu_V^v(\sigma^h+\mu^h)(\gamma^h+\mu^h)(\gamma^v+\mu_V^v)} \quad (3)$$

Supplementary Figure 1 : Summary of index P formulation, showing the climate-dependent and climate-independent parameters. Top: the R0 expression from which the suitability index is derived (expression 1) [1]. Middle: the R0 expression collapsed (expression 2). Bottom: the expression of the index P, highlighting in blue the parameters which are climate-dependent and which climatic variables each parameter is dependent on. Parameters not explicitly described in the figure are: σ^h host infectious period, μ^h host life span, γ^h host incubation period, $\phi^{h\to v}$ probability of transmission from host to vector upon bite on infected host, N number of hosts.

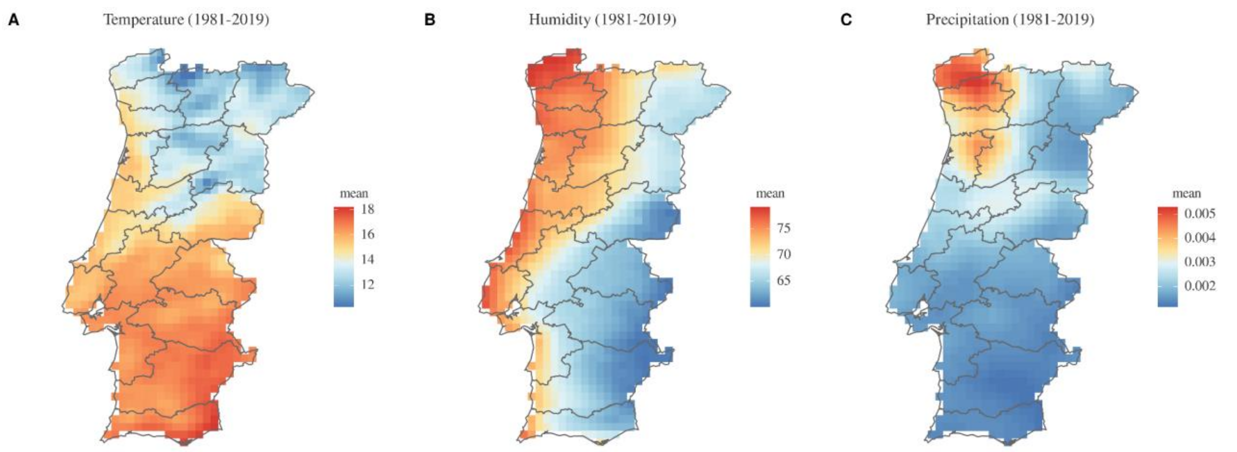

Supplementary Figure 2 : Summary of spatial distribution of meteorological variables in Portugal 1981-2019. Panel A shows the mean temperature, Panel B the mean humidity, and Panel C the mean precipitation over the entire time period using the climatic dataset described in the main text. Only temperature and humidity are used in the estimation of the suitability index presented in the main text (see Material and Methods in the main text).

Supplementary Tables

**Sample ***	**Type**	**N**	**Region**	**Period**	**Study**
Equine	VNT	7/24	Aljustrel	Summer 1970	[2]
Human	HI	38/1649	Country wide	1969 - 1973	[3](b)
Mammal	HI	16%	Portalegre, Évora, Beja	Sep 1966 - Feb 1967	[4](b)
Mammal	HI	3%	Portalegre, Évora, Beja	Sep 1966 - Feb 1967	[4](b)
Vector (*Culex pipiens* and *Cx. univitattus*)	PCR	pool	Ancão, Almancil	Jul 2004	[5](b)(c)
Vector (*Anopheles maculipennis*)	Isolation	pool	Barragem do Roxo	Sept 1969	[6]
Human	Serology	2	Faro	Jun - Jul 2004	[7]
Human	Serology	1	Setúbal	Jul 2010	[8]
Equine	VNT	40/1313	Santarém, Aveiro, Portalegre, Évora, Setúbal, Algarve	2004 - 2010	[9](a)
Avian (eagles, owls, storks, flamingos, ostriches, rheas and ibis)	VNT	23/116	Vila Real, Lisboa, Setúbal, Algarve	2004 - 2010	[9](a)
Equine	VNT	22/23	Loulé	Aug - Nov 2015	[10](a)
Equine	VNT	1/1	Faro	Aug - Oct 2015	[10](a)

Equine	VNT	1/1	Olhão	Sept 2015	[10](a)
Equine	VNT	8/9	Lagos	Sept - Oct 2015	[10](a)
Equine	VNT	4/4	Alcácer do Sal	Sept - Oct 2015	[10](a)
Equine	VNT	1/1	Arronches	Oct 2015	[10](a)
Equine	VNT	1/1	Alpiarça	Oct 2015	[10](a)
Equine	VNT	2/2	Loulé	Feb - Oct 2016	[10](a)
Equine	VNT	1/1	Lagos	Jul - Dec 2016	[10](a)
Equine	VNT	3/3	Silves	Oct - Dec 2016	[10](a)
Equine	VNT	1/1	Odemira	May 2016	[10](a)
Equine	VNT	1/1	Salvaterra de Magos	Jun - Dec 2016	[10](a)
Equine	VNT	2/2	Portalegre	Sep - Nov 2016	[10](a)
Equine	VNT	1/1	Alter-do-chão	Oct 2016	[10](a)
Equine	VNT	1/1	Benavente	Nov 2016	[10](a)
Equine	VNT	1/1	Elvas	Nov - Dec 2016	[10](a)
Equine	VNT	1/1	Beja	Nov - Dec 2016	[10](a)
Vector (*Culex pipiens* and *Cx. univitattus*)	PCR		Ria Formosa	Jul 2004	[11] (c)
Avian (*Accipiter gentilis*)	HI	1/4	Parque Nacional da Peneda-Gerês	1999 - 2002	[12](b)
Avian (*Bubo bubo*)	HI	1/19	Parque Natural do Vale do Guadiana	1999 - 2002	[12](b)
Avian (*Buteo buteo*)	HI	3/44	Parque Ecológico de Monsanto	1999 - 2002	[12](b)
Avian (*Ciconia ciconia*)	HI	2/12	Parque Ecológico de Monsanto	1999 - 2002	[12](b)
Avian (*Circaetus gallicus*)	HI	1/4	Parque Nacional da Peneda-Gerês	1999 - 2002	[12](b)
Avian (*Corvus corone*)	HI	1/4	Parque Ecológico de Monsanto	1999 - 2002	[12](b)
Avian (*Falco tinnunculus*)	HI	2/6	Parque Nacional da Peneda-Gerês	1999 - 2002	[12](b)
Avian (*Gyps fulvus*)	HI	1/3	Reserva Natural de Santo André	1999 - 2002	[12](b)

Avian (*Strix aluco*)	HI	1/3	Reserva Natural de Santo André	1999 - 2002	[12](b)
Avian (*Tyto alba*)	HI	3/13	Parque Nacional da Peneda-Gerês	1999 - 2002	[12](b)
Equine	Serology	3/41	Santarém	1999 - 2002	[12](b)
Human	VNT	1	Almancil	Jul 2015	[13]
Equine	Serology	1/18	Loulé	Aug 2015	[14]
Equine	Serology	1/54	Faro	Aug 2015	[14]
Equine	Serology	2/10	Loulé	Aug 2015	[14]
Avian (*Limosa limosa*)	ELISA	8/139	Reserva Natural de Santo André,Tagus estuary, Sado estuary	2006, 2008 - 2010	[15](b)
Avian (*Tringa totanus*)	ELISA	5/64	Reserva Natural de Santo André,Tagus estuary, Sado estuary	2006, 2008 - 2010	[15](b)
Avian (*Himantopus himantopus*)	ELISA	8/132	Reserva Natural de Santo André,Tagus estuary, Sado estuary	2006, 2008 - 2010	[15](b)
Equine	ELISA / PCR	23/165	Loulé, Portalegre, Silves, Alter-do-Chão, Lagoa, Elvas, Lagos, Benavente, Alcácer do Sal, Alcáçovas, Samora Correia, Albufeira, Escalos de Cima-Castelo Branco, Comporta	Aug 2016 - Sep 2020	This study

(a) Also IgM and prE-IgG serology, VNT only used when confirmed serology; (b) positive, implying Flavivírus cross-reactivity, but no WNV confirmed by other means; © Positive mosquito species report the same data; **HI:** hemagglutinin inhibition; **VNT:** virus neutralization test; **PCR:** polymerase chain reaction; **ELISA:** enzyme-linked immunosorbent assay.

Supplementary Table 1 - Sources including past evidence for WNV circulation in Portugal.

**Source**	**Region, period**	**Study**
Aedes berlandi, Ae. caspius, Anopheles atroparvus, An. claviger, Culex hortensis, Cx. laticinctus, Cx. mimeticus, Cx. pipiens, Cx. theileri, Cx. univittatus, Culiseta annulata, Cs. longiareolata, Cs. sunbochrea	Alqueva, monthly, 2004-2005, 2007	[16]
Aedes caspius, Ae. detritus, Anopheles atroparvus, Culex pipiens, Cx. theileri, Cx. univittatus, Culiseta annulata, Cs. longiareolata, Cs. subochrea	Comporta, monthly, 2004-2005	[16]
Aedes caspius, Ae, detritus, Anopheles atroparvus, An. algeriensis, Coquillettidia richiardii,	Algarve, monthly, Jun Sep 2007	[17]

Culex modestus, Cx. pipiens, Cx. theileri, Cx. univittatus, Culiseta longiareolata		
Aedes caspius, Culex pipiens	Sado estuary, Ria Formosa, monthly, May Oct 2005, 2006	[17]
Culex pipiens	Entire country, 2016 - 2019	REVIVE [18]

Supplementary Table 2 - Articles including time series of mosquito population dynamics in Portugal.

Location	Host	Genomic region	Year	Accession ID
Algarve	Mosquitoes	NS5	2004	AJ965631
Algarve	Mosquitoes	NS5	2004	AJ965627
Algarve	Mosquitoes	Polyprotein precursor	2004	AJ965630
Algarve	Mosquitoes	Polyprotein precursor	2004	AJ965629
Algarve	Mosquitoes	Polyprotein precursor	2004	AJ965628
Algarve	Mosquitoes	Polyprotein precursor	2004	AJ965626
Alentejo	Mosquitoes	Polyprotein precursor	1969	AM404308
Alentejo	Mosquitoes	Env	1969	AY727828

Supplementary Table 3 - Existing WNV genetic sequences from Portugal.

Supplementary References

1. Obolski, U. *et al.* MVSE: An R-package that estimates a climate-driven mosquito-borne viral suitability index. *Methods Ecol. Evol.* **10**, 1357–1370 (2019).
2. Filipe, A. R. & Campaniço, M. Encefalomielite equina por arbovírus. A propósito de uma epizootia presuntiva causada pelo vírus West Nile. *Revista Portuguesa de Ciências Veterinárias* **LXVIII**, (1973).
3. Filipe, A. R. Anticorpos contra arbovírus na população de Portugal. *Separata de O Médico* **LXVII**, 731–732 (1973).
4. Filipe, A. R. & Pinto, M. R. Survey for antibodies to arboviruses in serum of animals from southern Portugal. *Am. J. Trop. Med. Hyg.* **18**, 423–426 (1969).
5. Almeida, A. P. G. *et al.* Potential mosquito vectors of arboviruses in Portugal: species, distribution, abundance and West Nile infection. *Trans. R. Soc. Trop. Med. Hyg.* **102**, 823–832 (2008).
6. Filipe, A. R. Isolation in Portugal of West Nile virus from Anopheles maculipennis mosquitoes. *Acta Virol.* **16**, (1972).
7. Connell, J. *et al.* Two linked cases of West Nile virus (WNV) acquired by Irish tourists in the Algarve,

Portugal. *Weekly releases (1997–2007)* **8**, 2517 (2004).

8. Alves, M. J. *et al.* Infecção por vírus West Nile [Flavivírus] em Portugal. Considerações acerca de um caso clínico de síndrome febril com exantema. **8**, 46–51 (2012).
9. Barros, S. C. *et al.* Serological evidence of West Nile virus circulation in Portugal. *Vet. Microbiol.* **152**, 407–410 (2011).
10. Barros, S. C. *et al.* West Nile virus in horses during the summer and autumn seasons of 2015 and 2016, Portugal. *Vet. Microbiol.* **212**, 75–79 (2017).
11. Esteves, A. *et al.* West Nile virus in Southern Portugal, 2004. *Vector Borne Zoonotic Dis.* **5**, (2005).
12. Formosinho, P. *et al.* O vírus West Nile em Portugal – estudos de vigilância epidemiológica. *Revista Portuguesa de Ciências Veterinárias* **101**, 61–68 (2006).
13. Zé-Zé, L. *et al.* Human case of West Nile neuroinvasive disease in Portugal, summer 2015. *Euro Surveill.* **20**, (2015).
14. World Organization for Animal Health (OIE) - West Nile reports. *Information received on 03/09/2015 from Prof. Dr Álvaro Mendonça, Director General, Direcção Geral de Alimentação e Veterinária, Ministério da Agricultura E do Mar, Lisboa, Portugal (2015)* https://www.oie.int/wahis_2/public/wahid.php/Reviewreport/Review?page_refer=MapFullEventReport&reportid=18585.
15. Pardal, S. *et al.* Shorebird low spillover risk of mosquito-borne pathogens on Iberian wetlands. *J. Ornithol.* **155**, 549–554 (2013).
16. Almeida, A. P. G. *et al.* Mosquito surveys and West Nile virus screening in two different areas of southern Portugal, 2004-2007. *Vector Borne Zoonotic Dis.* **10**, 673–680 (2010).
17. Alves, J. M. *et al.* Flavivírus transmitidos por mosquitos: um risco potencial para Portugal. *Investigação em ambiente e saúde - desafios e estratégias (Universidade de Aveiro)* (2009).
18. REVIVE - Rede de Vigilância de Vetores. http://www2.insa.pt/sites/INSA/Portugues/AreasCientificas/DoencasInfecciosas/AreasTrabalho/EstVectDoencasInfecciosas/Paginas/Revive.aspx.
